# Temporal trends in clozapine use at time of discharge among people with schizophrenia at two public psychiatric hospitals in Taiwan, 2006–2017

**DOI:** 10.1038/s41598-020-75022-8

**Published:** 2020-10-22

**Authors:** Ching-Hua Lin, Hung-Yu Chan, Chun-Chi Hsu, Feng-Chua Chen

**Affiliations:** 1 Kaohsiung Municipal Kai-Syuan Psychiatric Hospital, Kaohsiung, Taiwan; 2 Department of Psychiatry, School of Medicine, College of Medicine, Kaohsiung Medical University, Kaohsiung, Taiwan; 3 Department of General Psychiatry, Taoyuan Psychiatric Center, No. 71, Long-Show Street, Taoyuan City, 33058 Taiwan; 4 Department of Psychiatry, National Taiwan University Hospital and College of Medicine, National Taiwan University, Taipei, Taiwan

**Keywords:** Psychology, Diseases, Medical research

## Abstract

Clozapine treatment remains the gold standard for treatment-resistant schizophrenia. This study aimed to describe temporal trends in clozapine use at discharge among patients with schizophrenia at two of the largest public psychiatric hospitals in Taiwan over a twelve-year period. Patients with schizophrenia discharged from the two study hospitals between 2006 and 2017 (n = 24,101) were included in the analysis. Antipsychotic augmentation was defined as concomitant use of a second antipsychotic as augmentation to clozapine treatment. Changes in the rate of clozapine use and antipsychotic augmentation at discharge over time were analyzed using the Cochran-Armitage trend test. Patients discharged on clozapine had significantly longer hospital stays than other patients. The rate of clozapine use at discharge increased from 13.8% to 20.0% over time (Z = 6.88, p < .0001). Concomitant use of anticholinergic medication was more common in patients receiving antipsychotic augmentation than clozapine antipsychotic monotherapy. Among patients discharged on clozapine, the rate of augmentation with a second antipsychotic increased from 19.1% to 36.2% over time (Z = 6.58, p < .0001). Among patients receiving antipsychotic augmentation, use of another second-generation antipsychotic as the augmentation agent grew from 32.6% to 65.5% over time (Z = 8.90, p < .0001). The increase in clozapine use was accompanied by an increase in concomitant use of a second antipsychotic as augmentation during the study period. Further studies are warranted to clarify the risk/benefit of this augmentation strategy. Clozapine may still be underutilized, and educational programs are needed to promote clinical use of clozapine.

## Introduction

With a worldwide prevalence of about 0.5–0.7%, schizophrenia is a common psychiatric disorder^1,2^, and antipsychotic medications are the cornerstone for the treatment of schizophrenia. However, treatment resistance to antipsychotic medications has been observed in approximately one-third of patients with schizophrenia^3^. Treatment-resistant schizophrenia (TRS) is commonly defined as lack of response to two or more antipsychotic trials of adequate dose (i.e., at least 600 mg chlorpromazine equivalent per day) and duration (i.e., at least 6 weeks)^4^. In comparison with other patients with schizophrenia, patients with TRS experience poorer quality of life, more comorbidities, and higher medical expenditures^5^.

Many agree clozapine is by far the most effective treatment for TRS^6–8^. Studies have shown it to be associated with significantly fewer extrapyramidal side effects which may account for the reduced treatment discontinuation rates^9^. Lower mortality rates, hospitalization rates and suicide risk are also seen^6,10–12^. Unfortunately, clozapine is often underused in patients with TRS despite its proven efficacy. Potential adverse effects, such as metabolic syndrome, bowel obstruction, agranulocytosis, pneumonia, and myocarditis may have limited its use^13–15^. With the aforementioned rate of treatment resistance in patients with schizophrenia, it is reasonable to expect that around 30% of the patients would be treated with clozapine, but this is far from reality. For example, 6.7% of patients with schizophrenia are treated with clozapine in Quebec^16^, 10.2% in Denmark^17^, 12.7% in India, 11% in Hong Kong^18^, and 23% in England and Wales^19,20^. Additionally, inadequate doses and treatment delays have also been reported^6,21^.

Nonetheless, not all patients with TRS will respond to clozapine antipsychotic monotherapy. It is estimated that approximately 40–70% of patients with TRS do not respond to clozapine antipsychotic monotherapy of adequate dose and duration^22,23^. In the absence of a clear consensus on what strategies to implement when facing clozapine failure, augmentation with a second antipsychotic is common in clinical practice^22^. The strategy of augmentation endeavors to enhance efficacy through exploiting different receptor-binding profiles among antipsychotics. Case in point, since clozapine has a lower affinity for dopamine D2 receptors and a faster dissociation rate from D2 receptors^24^, clozapine in combination with a second antipsychotic agent with a higher affinity for D2 receptors may enhance the efficacy^25^. Currently, in the event of clozapine antipsychotic monotherapy failure, rates of augmentation with a second antipsychotic agent fluctuate from 18 to 44%^26,27^.

The aim of this study was to illustrate the temporal trends in clozapine use and augmentation with a second antipsychotic to clozapine treatment at time of discharge among patients with schizophrenia discharged from two public psychiatric hospitals in Taiwan over a twelve-year period (2006–2017).

## Methods

### Ethics

The two public hospitals in southern and northern Taiwan respectively, Kaohsiung Municipal Kai-Syuan Psychiatric Hospital (KSPH) and Taoyuan Psychiatric Center (TYPC), chosen for this study have the largest numbers of acute care psychiatric beds out of all the institutes in Taiwan. There are 7296 acute care psychiatric beds in Taiwan and the number of acute ward beds in the two study hospital is 786 (10.8%, 786/7296). It is our hope that the large sample size and its multi-regional (different areas of Taiwan) source would improve the representativeness of the study sample. The study was approved by the institutional review boards of both hospitals and was carried out in accordance with the Declaration of Helsinki (2013) as well as Taiwan’s national legislation (Human Subjects Research Act, Taiwan). Informed consents were waived by the ethics committees of the two study hospitals (Ethics Committee of Taoyuan Psychiatric Center and Institutional Review Board of Kaohsiung Municipal Kai-Syuan Psychiatric Hospital) as all data collected were part of routine clinical care and anonymized prior to analysis.

### Subjects and design

Patients were considered eligible if they were discharged from an acute care ward to the community on antipsychotics between January 2006 and December 2017 with a DMS-IV-TR or DMS-5 diagnosis of schizophrenia or schizoaffective disorder^28,29^. Diagnoses were made by board-certified psychiatrists on the basis of clinical observations and interviews made during hospitalization, previous medical records and information provided by the patient’s primary caregiver. Patient data were retrospectively extracted from the medical records and the electronic health information system. Patients who did not have complete prescription data available or were in a clinical trial during the study period were excluded. The primary outcome measure was the antipsychotic medications the patient was on at discharge. As-needed (p.r.n.) medications were excluded from the analysis. As patients were usually discharged after a period of stabilization, these were the medications that the patient stabilized on. The antipsychotics used were grouped into first-generation (FGAs) and second-generation antipsychotics (SGAs). SGAs available at the two hospitals during the study period included risperidone, amisulpride, aripiprazole, clozapine, olanzapine, paliperidone, quetiapine, sulpiride^30–32^, ziprasidone and zotepine^33^.

### Statistical analysis

Data were analyzed using SPSS version 17.0 for Windows (SPSS Inc., Chicago, IL, USA) and SAS 9.3 software (SAS institute Inc., Cary, North Carolina). Statistical significance was defined as a p-value < 0.05. The Pearson’s chi-squared test and the independent t test were used to compare demographic and clinical characteristics between groups.

The Cochran-Armitage trend test was used to evaluate whether statistically significant temporal trends existed for clozapine prescription rates between 2006 and 2017. For patients discharged on clozapine, we also performed the Cochran-Armitage trend test to evaluate whether statistically significant temporal trends existed for rates of augmentation with a second antipsychotic (FGA or SGA) among patients treated with clozapine.

In order to validate the primary analysis examining trends in clozapine use and clozapine antipsychotic augmentation at discharge, three sensitivity analyses were conducted^34^. In the first analysis, for patients with multiple hospitalizations during the study period only the last hospitalization was included. This is due to the fact that clozapine is often viewed as a “drug of last resort” in clinical practice^35^. For the second and third analysis, only the patients discharged from a single study hospital, KSPH and TYPC respectively, were included.

### Ethical standards

The study was approved by the institutional review boards (IRB) of both Kaohsiung Municipal Kai-Syuan Psychiatric Hospital and Taoyuan Psychiatric Center and was carried out in accordance with the Declaration of Helsinki (2013) as well as Taiwan’s national legislation (Human Subjects Research Act). IRB Number: B20170929.

### Informed consent

Informed consents were not acquired as all data collected were part of routine clinical care and anonymized prior to analysis.

## Results

### Descriptive statistics

A total of 24, 840 patients with schizophrenia or schizoaffective disorder were discharged from the two study hospitals during the study period, and 24,101 were included in the analysis. Table 1 lists the demographic and clinical characteristics of these patients. Among the 24,101 patients included in the analysis, 4248 (17.6%) were discharged on clozapine. Eleven thousand, three hundred and sixty-one (47.1%) were male, and 12,740 (52.9%) were female. The mean age was 41.4 ± 12.2 years.

**Table 1:** Demographic and clinical characteristics of the study population.

	All patients (n = 24,101)		KSPH^a^ patients (n = 13,476)		TYPC^b^ patients (n = 10,625)		*P*^c^
	n	%	n	%	n	%	
Sex							** < 0.001 **
Male	11,361	47.1	5811	43.1	5550	52.2	
Female	12,740	52.9	7665	56.9	5075	47.8	
Discharged on clozapine							** < 0.001 **
Yes	4248	17.6	2249	16.7	1999	18.8	
No	19,853	82.4	11,227	83.3	8626	81.2	

	Mean	SD	Mean	SD	Mean	SD	P^d^
Age (years)	41.4	12.2	43.2	11.8	39.2	12.2	** < 0.001**
Length of hospital stay (days)	179.3	446.2	173.5	386.5	186.6	512.0	**0.024**
Clozapine daily dose (mg)	268.4	138.2	234.1	120.8	307.0	146.2	** < 0.001**

Bold, statistically significant.

^a^KSPH = Kai-Syuan Psychiatric Hospital.

^b^TYPC = Taoyuan Psychiatric Center.

^c^Pearson’s χ^2^ test.

^d^Independent t test.

There were significant differences between patients discharged from the two study hospitals on several variables (Table 1). Patients discharged from KSPH were older and more likely to be female. In addition, they had a shorter length of hospital stay and were less likely to be on clozapine at discharge. Among patients receiving clozapine treatment, the daily dose was significantly lower in those discharged from KSPH.

### Patients discharged on clozapine versus others

Comparisons between patients discharged on clozapine and others in demographic and clinical characteristics are shown in Table 2. There was no statistically significant difference between the two groups with respect to sex or age. Compared with other patients, patients discharged on clozapine had significantly longer hospital stays. Table 3 lists the percentage of patients discharged on clozapine each year between 2006 and 2017. The results of the Cochrane-Armitage trend test showed a significant increasing trend in clozapine use at discharge (Z = 6.88, p < 0.0001). Supplemental Fig. 1 illustrates temporal trends in clozapine use at discharge among KSPH, TYPC, and all patients.

**Table 2:** Comparisons between patients discharged on clozapine and others.

	Patients discharged on clozapine (n = 4248)		Others (n = 19853)		p^a^
	n	%	n	%	
Sex					0.230
Male	1967	46.3	9394	47.3	
Female	2281	53.7	10459	52.7	

	Mean	SD	Mean	SD	p^b^
Age (years)	41.6	11.0	41.4	12.4	0.302
Length of hospital stay (days)	350.5	672.6	142.7	370.5	**< 0.001**

Bold, statistically significant

^a^Pearson’s χ^2^ test

^b^Independent t test.

**Table 3:** Percentage of patients discharged on clozapine, 2006–2017.

Year	2006	2007	2008	2009	2010	2011
No. of patients discharged on clozapine (%)	241 (13.8%)	287 (15.1%)	321 (15.2%)	342 (16.8%)	363 (17.4%)	345 (17.1%)
No. of patients discharged	1752	1898	2113	2035	2088	2021
Year	2012	2013	2014	2015	2016	2017
No. of patients discharged on clozapine (%)	410 (19.4%)	395 (19.6%)	420 (20.2%)	346 (17.3%)	386 (19.1%)	392 (20.0%)
No. of patients discharged	2117	2015	2077	2004	2024	1957

### Clozapine antipsychotic monotherapy versus augmentation with a second antipsychotic

Among the 4,248 patients discharged on clozapine, 1,374 (32.3%) were also prescribed a second antipsychotic as augmentation to clozapine treatment. Antipsychotics used for augmentation are listed in Supplementary Table S1 online. In contrast, 2,874 (67.7%) patients received clozapine antipsychotic monotherapy. Table 4 lists the differences in demographic and clinical characteristics between the two groups. The female gender was overrepresented in patients receiving augmentation therapy. Patients receiving augmentation therapy were also more likely to be on an anticholinergic agent and received lower doses of clozapine. However, the observed differences in clozapine dose between the two groups were quite small. No statistically significant differences existed between the two groups in terms of age or length of hospital stay. The Cochrane-Armitage trend test results revealed that there was a significant increase in using a second antipsychotic as augmentation among patients discharged on clozapine during the study period (Z = 6.58, p < 0.0001).

**Table 4:** Comparisons between patients receiving augmentation therapy and antipsychotic monotherapy among patients discharged on clozapine.

	Augmentation therapy n = 1374)		Antipsychotic monotherapy (n = 2874)		p^a^
	n	%	n	%	
Sex					** < 0.001**
Male	560	40.8	1407	49.0	
Female	814	59.2	1467	51.0	
Use of anticholinergics					** < 0.001**
Yes	665	48.4	658	22.9	
No	709	51.6	2216	77.1	

	Mean	SD	Mean	SD	p^b^
Age (years)	41.3	10.9	41.8	11.1	0.230
Length of hospital stay (days)	330.7	636.2	360.0	689.3	0.184
Clozapine daily dose (mg)	261.3	155.5	271.8	129.0	**0.021**

Bold, statistically significant.

^a^Pearson’s χy^2^ test.

^b^Independent t test.

### Clozapine + another SGA versus clozapine + an FGA

Among the 1,374 patients receiving a second antipsychotic as augmentation to clozapine treatment, another SGA was used in 668 (48.6%), while an FGA was used in 706 (51.4%). Patients who received augmentation with another SGA were older and less likely to be on anticholinergics (Supplementary Tables S1& S1 online). There was also a significant increase in using another SGA as the augmentation agent, going from 32.6% to 65.5% over the study period (Z = 8.90, p < 0.0001) among patients receiving augmentation therapy. The rates of antipsychotic augmentation among patients discharged on clozapine and the rates of using another SGA as the augmentation agent are shown in Table 5.

**Table 5:** Rate of augmentation with a second antipsychotic among patients discharged on clozapine and rate of SGA^a^ augmentation among all augmentation patients, 2006–2017.

Year	2006	2007	2008	2009	2010	2011
No. of patients discharged on clozapine + a second antipsychotic (%)	46 (19.1)	70 (24.4)	90 (28.0)	104 (30.4)	123 (33.9)	121 (35.1)
No. of patients discharged on clozapine + a second SGA^a^ (%)	15 (32.6%)	26 (37.1%)	24 (26.7%)	32 (30.8%)	44 (35.8%)	54 (44.6%)
No. of patients discharged on clozapine	241	287	321	342	363	345

Year	2012	2013	2014	2015	2016	2017
No. of patients discharged on clozapine + a second antipsychotic (%)	107 (26.1)	126 (31.9)	146 (34.8)	148 (42.8)	151 (39.1)	142 (36.2)
No. of patients discharged on clozapine + a second SGA^b^ (%)	54 (50.5%)	66 (52.4%)	76 (52.1%)	86 (58.1%)	98 (64.9%)	93 (65.5%)
No. of patients discharged on clozapine	410	395	420	346	386	392

^a^FGA = Firs-generation antipsychotic.

^b^SGA = Second-generation antipsychotic.

### Sensitivity analyses

The results of the sensitivity analyses were consistent with the primary analysis. When only the last hospitalization of each subject was included in the analysis, the results still showed significant increasing trends in clozapine use at discharge (Z = 4.60, p < 0.0001) (Supplementary Table S3 online) and use of a second antipsychotic as augmentation to clozapine therapy (Z = 4.36, p < 0.0001) (Supplementary Table S4 online). In addition, when only patients from a single study hospital, KSPH and TYPC respectively, were included in the analysis, both hospitals saw significant increases in clozapine use (Z = 5.14, p < 0.0001 for KSPH; Z = 4.75, p < 0.0001 for TYPC) (Supplementary Tables S5 & S7 online) and use of a second antipsychotic as augmentation (Z = 4.63, p < 0.0001 for KSPH; Z = 4.79, p < 0.0001 for TYPC) (Supplementary Tables S6 & S8 online).

## Discussion

The main finding of this study was that the percentage of patients discharged on clozapine increased from 13.8% to 20.0% between 2006 and 2017. This is consistent with the results of the study by Bachmann et al.^36^. They concluded a rise in clozapine prescription was seen in most countries from 2005 to 2014. This boost perhaps reflects the gained experience and success with this drug among clinicians in treating patients with TRS. Keeping in mind that treatment resistance is seen in up to a third of schizophrenia patients^3^, it stands to reason that around 30% of schizophrenia patients should be prescribed clozapine. In addition, clozapine is the only antipsychotic proven effective for recurrent suicidal behavior in schizophrenia patients^37^. Therefore, with an average prescription rate of only 17.6%, clozapine may have been underutilized at the two study hospitals.

The many possible adverse effects of clozapine have often led it to be seen as a last resort. Clinicians generally go through multiple failed trials of other antipsychotics prior to initiation of clozapine^35^. This could be an explanation for why patients discharged on clozapine had longer hospital stays in this study (Table 2).

In the current study, length of hospital stay (179.3 ± 446.2 days) (Table 1) was extended for all patients with schizophrenia. This may be a direct result of the reality that community-based mental health services are still somewhat under-developed in Asia, and long-term institutionalization of psychiatric patients is more common than in western countries^38^. For example, the average length of stay for psychiatric hospitalization in Japan was 274 days in 2018^39^.

According to the World Health Organization, the defined daily dose for clozapine is 300 mg/d^40,41^, which is higher than the average clozapine dose (268.4 ± 138.2 mg) observed in this study (Table 1). One possible explanation for this phenomenon is that metabolism of clozapine may be slower in Asians^42,43^. Whether such dosages are adequate in Asian patients could be confirmed by future studies focused on plasma clozapine levels^4^. Furthermore, the prevalence of cigarette smoking and nicotine replacement therapy among psychiatric inpatients in Taiwan is unlike other countries^44^. The results of a meta-analysis of studies across 20 nations showed the prevalence of current smoking in patients with schizophrenia was 62%^45^. However, in Taiwan the prevalence of current smoking in patients with schizophrenia was only 38.1%^46^ which was considerably lower. Previous studies showed that clozapine plasma concentration levels are 35–48% lower in cigarette smokers compared to non-smokers^47^. These observations could be the reason behind the lower average dose of clozapine seen in this study.

We also found the proportion of clozapine-treated patients receiving augmentation with a second antipsychotic had steadily increased throughout the study period, going from 19.1% to 36.2% (Table 5). The percentage of patients receiving antipsychotic augmentation in this study, i.e., 32.3%, was in the same ballpark as a UK study that declared 30%^48^. By the end of the study period, the percentage of patients using a second SGA as the augmentation agent among all augmentation patients had also increased significantly (32.2–65.5% ) (Table 5). This finding may be interpreted in several different ways.

It may reflect the fact that many patients treated with clozapine are clinically complex which necessitates treatment with high doses and polypharmacy, sometimes at the expense of increased side-effect burden^49^. Furthermore, chronicity, treatment resistance and comorbid conditions are often more common among patients in public institutions^50^. In real-world clinical practice, especially in a setting such as public psychiatric hospitals, from time to time clinicians will encounter schizophrenia patients with prominent impulsivity and aggression that simply do not respond to clozapine. Under these circumstances, augmentation with a second antipsychotic may be the only viable option even though there is currently no consensus regarding such a strategy^22^.

As clozapine in some patients may take longer than the usual 4–8 weeks to achieve clinical response^49,51^, clinicians may resort to augmentation with a second antipsychotic to speed up symptom reduction in order to satisfy patient expectations and healthcare policies.

It must be said that the decision to use an augmentation agent is not always due to incomplete response to clozapine. In this study, the average clozapine dose used in patients receiving augmentation therapy (261.8 mg/d) (Table 4) was considerably lower than the recommended threshold for establishing non-response to clozapine treatment. When plasma clozapine levels are unavailable, Howes and colleagues^4^ recommend that a minimum dosage of 500 mg/d should be tried for at least 3 months prior to establishing treatment resistance to clozapine. Clinicians are often hesitant to use higher doses of clozapine because of dose-related adverse effects, such as seizure, sedation, tachycardia, orthostatic hypotension, nocturnal enuresis and sialorrhea^52,53^. Augmentation strategy may be adopted with the expectation to reduce symptom severity without increasing the clozapine dose, thus avoiding dose-related adverse effects of clozapine^49^. However, compared with patients receiving clozapine antipsychotic monotherapy, concomitant use of anticholinergics was more common in those receiving antipsychotic augmentation (Table 4). Anticholinergic agents are generally used to treat extrapyramidal side effects (EPSE). It is possible that the augmentation strategy causes more EPSE than clozapine antipsychotic monotherapy. In addition, clozapine itself also has significant anticholinergic properties. This finding is clinically relevant because antipsychotic augmentation may lead to more anticholinergic side effects, such as dry mouth, constipation, urinary retention, and cognitive impairment. Finally, different individuals may exhibit highly diverse responses to augmentation with a second antipsychotic. Some clozapine partial responders may indeed benefit from such augmentation strategy^54^.

In this study, use of a second SGA as the augmentation agent to clozapine treatment steadily became more common among patients receiving antipsychotic augmentation (Table 5). Use of anticholinergics was less common in patients receiving SGA augmentation than those receiving FGA augmentation (Table S2). Additionally, the rate of SGA augmentation increased gradually during the study period (Table 5). One possibility is that SGAs may cause fewer EPSE than FGAs, thus are more appealing to patients and clinicians as an augmentation agent to clozapine^55^.

To the best of our knowledge, this is the first study to investigate temporal trends in both clozapine use among patients with schizophrenia and the rates of antipsychotic augmentation among patients on clozapine. The results could help paint a clearer picture regarding clinical use of clozapine. Another strength of our study was that given the large sample size (n = 24,101) there was enough statistical power to help reduce type II error. Additionally, use of an inpatient-only sample ensured medication adherence as it was reliably monitored by the nursing staff.

However, some limitations should be considered while interpreting the results of this study. First, this was a study of retrospective design, and the sample was not randomized. Second, we did not control for the effects of concomitant use of other psychotropics that may be relevant to the analysis, including antidepressants, mood stabilizers and glutamatergic agents^56^. Third, this was a hospital-based study, and only inpatients were included. As inpatients are prone to be more severely/chronically ill and/or treatment-resistant, the findings of this study may not be generalizable to outpatients. Fourth, as both of the study sites were large public psychiatric hospitals, generalizability of the findings to general hospitals may be limited. Fifth, it is possible patients were in the process of cross tapering from a previous antipsychotic to clozapine at discharge, and the study design did not take this into account. However, the number of these patients would be minimal as it is common practice at both study hospitals to discharge patients only after reaching the maintenance phase of treatment^57^. The extended length of hospital stay for patients discharged on clozapine (350.5 ± 672.6 days) (Table 1) also lends support to this line of thinking. Sixth, in the analysis patients were counted multiple times if they had multiple hospitalizations during the study period. In addition, there were significant differences in some clinical characteristics between patients at the two study hospitals (Table 1). These were all potential confounders for the study. However, the three sensitivity analyses consistently demonstrate that both clozapine use and clozapine antipsychotic augmentation increased significantly during the study period, same as the primary analysis. Therefore, results of the primary analysis remained robust despite these potential confounders. Lastly, data on some important clinical characteristics were not collected in this study, e.g., treatment duration, symptom severity, functional impairment, risk of suicide or violence, adverse events and comorbidity.

## Conclusion

In conclusion, we found that clozapine was still underused, and when used it was often at an inadequate dosage. Additionally, patients discharged on clozapine had longer hospital stays than other patients. Educational programs for professionals, patients and family members are vital to promote proper use of clozapine^58^. More studies are needed to address the implementation of effective strategies to facilitate clinical use of clozapine. The surge in clozapine use went hand in hand with an increase in the percentage of patients receiving augmentation with a second antipsychotic. Antipsychotic augmentation has grown in popularity, especially strategies that involve a second SGA. However, concomitant use of anticholinergics was more common in patients receiving antipsychotic augmentation than those receiving antipsychotic monotherapy. Further research is warranted to examine the relationship between risks and benefits and also the cost-effectiveness of this augmentation strategy.

## Supplementary information

Supplementary Information 1.

Supplementary Information 2.

Supplementary Information 3.
