# Supplementary material for: Temporal trends in clozapine use at time of discharge among people with schizophrenia at two public psychiatric hospitals in Taiwan, 2006–2017
Source: Sci Rep. 2020 Oct 22;10:17984. doi: 10.1038/s41598-020-75022-8 (PMC7581717; doi:10.1038/s41598-020-75022-8)
Supplement: Supplementary file 1 — Supplementary Information 1. [file 41598_2020_75022_MOESM1_ESM.docx]

**Title of manuscript**

Temporal trends in clozapine use at time of discharge among people with schizophrenia at two public psychiatric hospitals in Taiwan, 2006-2017

**Names of authors**

Ching-Hua Lin, M.D., Ph.D., Hung-Yu Chan, M.D., Ph.D., Chun-Chi Hsu, M.D., Feng-Chua Chen, B.S.

Supplementary Table S1. Antipsychotics used for augmentation

Supplementary Table S2. Comparisons between patients discharged on clozapine + another SGA vs. clozapine + an FGA

Supplementary Table S3. Percentage of patients discharged on clozapine, 2006-2017 (For patients with multiple hospitalizations, only the last hospitalization was included in the analysis)

Supplementary Table S4. Rate of augmentation with a second antipsychotic among patients discharged on clozapine, 2006-2017 (For patients with multiple hospitalizations, only the last hospitalization was included in the analysis)

Supplementary Table S5. Percentage of patients discharged on clozapine, 2006-2017 (Only patients discharged from KSPH were included in the analysis)

Supplementary Table S6. Rate of augmentation with a second antipsychotic among patients discharged on clozapine, 2006-2017 (Only patients discharged from KSPH were included in the analysis)

Supplementary Table S7. Percentage of patients discharged on clozapine, 2006-2017 (Only patients discharged from TYPC were included in the analysis)

Supplementary Table S8. Rate of augmentation with a second antipsychotic among patients discharged on clozapine, 2006-2017 (Only patients discharged from TYPC were included in the analysis)

Supplementary Fig. S1. Clozapine prescription rates among schizophrenia patients at discharge, 2006-2017
