# Supplementary figures and images for: Temporal trends in clozapine use at time of discharge among people with schizophrenia at two public psychiatric hospitals in Taiwan, 2006–2017
Source: Sci Rep. 2020 Oct 22;10:17984. doi: 10.1038/s41598-020-75022-8 (PMC7581717; doi:10.1038/s41598-020-75022-8)

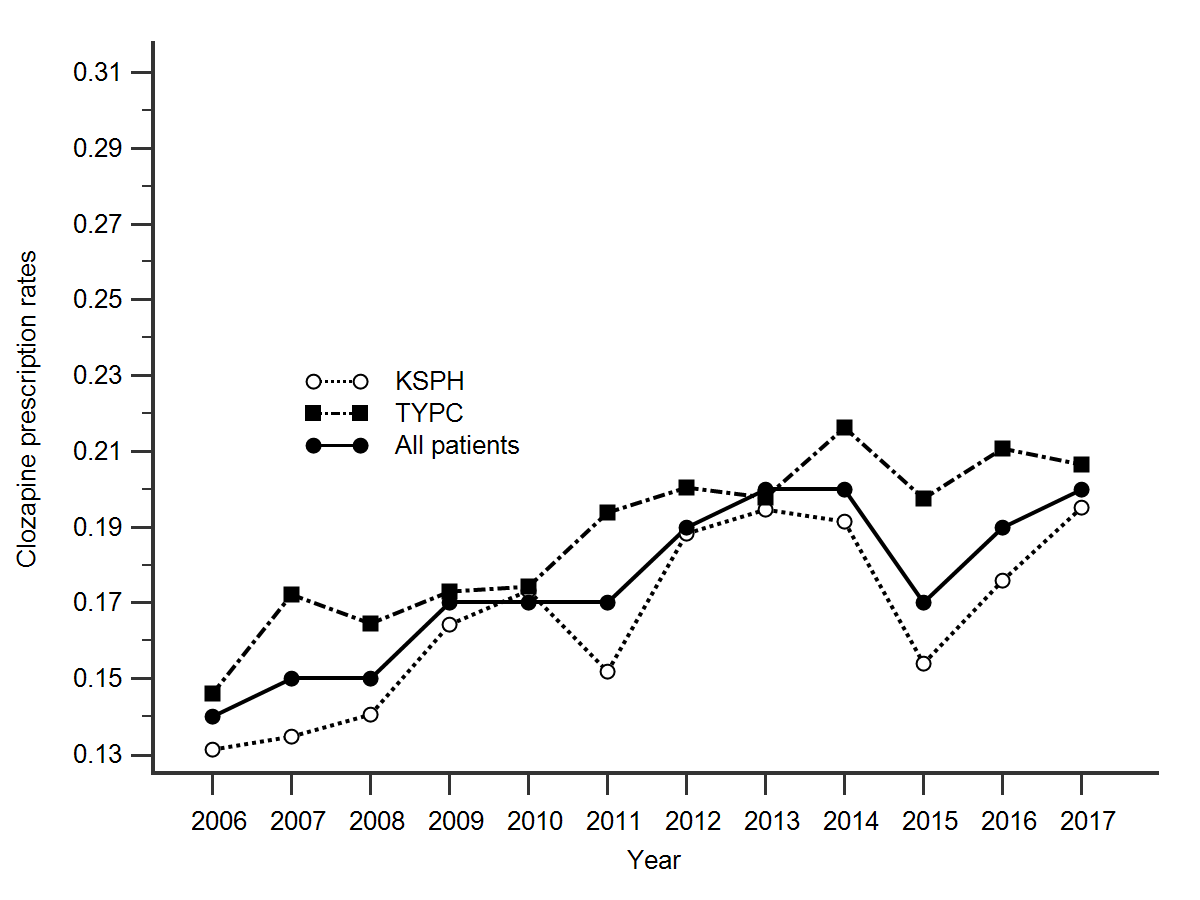

Supplement: Supplementary file 2 — Supplementary Information 2. [file 41598_2020_75022_MOESM2_ESM.tif]
