# Supplementary material for: Temporal trends in clozapine use at time of discharge among people with schizophrenia at two public psychiatric hospitals in Taiwan, 2006–2017
Source: Sci Rep. 2020 Oct 22;10:17984. doi: 10.1038/s41598-020-75022-8 (PMC7581717; doi:10.1038/s41598-020-75022-8)
Supplement: Supplementary file 3 — Supplementary Information 3. [file 41598_2020_75022_MOESM3_ESM.docx]

Supplementary Table S1. Antipsychotics used for augmentation

| Classification | Antipsychotic | n | Mean dose (mg/d) ± SD |
| --- | --- | --- | --- |
| FGA^1^ | chlorpromazine | 130 | 189.42 ± 138.82 |
|  | clopenthixol | 1 | 14.29 |
|  | clotiapine | 36 | 66.11 ± 43.77 |
|  | flupentixol | 42 | 3.09 ± 3.02 |
|  | haloperidol | 478 | 8.03 ± 6.21 |
|  | loxapine | 2 | 50.00 ± 0.00 |
|  | thioridazine | 9 | 233.33 ± 96.82 |
|  | trifluoperazine | 8 | 22.50 ± 10.35 |
| SGA^2^ | amisulpride | 19 | 447.37 ± 335.61 |
|  | aripiprazole | 35 | 17.43 ± 8.71 |
|  | olanzapine | 53 | 13.96 ± 8.05 |
|  | paliperidone | 22 | 11.32 ± 4.13 |
|  | quetiapine | 25 | 339.00 ± 269.56 |
|  | risperidone | 137 | 3.24 ± 1.54 |
|  | sulpiride | 351 | 589.89 ± 384.3 |
|  | ziprasidone | 5 | 116.00 ± 35.78 |
|  | zotepine | 21 | 114.29 ± 70.52 |

^1^FGA = First generation antipsychotic

^2^SGA = Second generation antipsychotic

Supplementary Table S2. Comparisons between patients discharged on clozapine + another SGA vs. clozapine + an FGA

|  |  | clozapine + another SGA  (n = 668) | |  | clozapine + an FGA  (n = 706) | |  |  |
| --- | --- | --- | --- | --- | --- | --- | --- | --- |
|  |  | n | % |  | n | % |  | p ^1^ |
| Sex  Male  Female |  | 284  384 | 42.5  57.5 |  | 276  430 | 39.1  60.9 |  | 0.197 |
| Use of anticholinergics  Yes  No |  | 304  364 | 45.5  54.5 |  | 361  345 | 51.1  48.9 |  | **0.037** |
|  |  | Mean | SD |  | Mean | SD |  | p ^2^ |
| Age (years) |  | 42.7 | 11.5 |  | 40.1 | 10.0 |  | **<0.001** |
| Length of hospital stay (days) |  | 332.1 | 668.4 |  | 329.3 | 604.6 |  | 0.934 |
| Clozapine daily dose (mg) |  | 261.9 | 166.9 |  | 260.7 | 144.1 |  | 0.881 |

**Bold,** statistically significant

^1^ Pearson’s χ^2^ test

^2^ Independent t test

Supplementary Table S3. Percentage of patients discharged on clozapine, 2006-2017 (For patients with multiple hospitalizations, only the last hospitalization was included in the analysis)

| Year | 2006 | 2007 | 2008 | 2009 | 2010 | 2011 |
| --- | --- | --- | --- | --- | --- | --- |
| No. of patients discharged on clozapine (%) | 66  (13.3%) | 90  (16.8%) | 86  (13.2%) | 89  (15.1%) | 85  (14.2%) | 101  (16.5%) |
| No. of patients discharged | 496 | 535 | 653 | 588 | 598 | 612 |
| Year | 2012 | 2013 | 2014 | 2015 | 2016 | 2017 |
| No. of patients discharged on clozapine (%) | 125  (19.2%) | 124  (19.8%) | 148  (19.8%) | 146  (17.4%) | 184  (18.2%) | 294  (19.7%) |
| No. of patients discharged | 652 | 627 | 749 | 839 | 1013 | 1494 |

Supplementary Table S4. Rate of augmentation with a second antipsychotic among patients discharged on clozapine, 2006-2017 (For patients with multiple hospitalizations, only the last hospitalization was included in the analysis)

| Year | 2006 | 2007 | 2008 | 2009 | 2010 | 2011 |
| --- | --- | --- | --- | --- | --- | --- |
| No. of patients discharged on clozapine + a second antipsychotic (%) | 15  (22.7%) | 17  (18.9%) | 23  (26.7%) | 20  (22.5%) | 24  (28.2%) | 28  (27.7%) |
| No. of patients discharged on clozapine | 66 | 90 | 86 | 89 | 85 | 101 |
| Year | 2012 | 2013 | 2014 | 2015 | 2016 | 2017 |
| No. of patients discharged on clozapine + a second antipsychotic (%) | 29  (23.2%) | 43  (34.7%) | 48  (32.4%) | 54  (37.0%) | 62  (33.7%) | 107  (36.4%) |
| No. of patients discharged on clozapine | 125 | 124 | 148 | 146 | 184 | 294 |

Supplementary Table S5. Percentage of patients discharged on clozapine, 2006-2017 (Only patients discharged from KSPH^1^ were included in the analysis)

| Year | 2006 | 2007 | 2008 | 2009 | 2010 | 2011 |
| --- | --- | --- | --- | --- | --- | --- |
| No. of patients discharged on clozapine (%) | 134  (13.1%) | 143  (13.5%) | 157  (14.1%) | 189  (16.4%) | 195  (17.3%) | 169  (15.2%) |
| No. of patients discharged | 1020 | 1062 | 1117 | 1151 | 1125 | 1113 |
| Year | 2012 | 2013 | 2014 | 2015 | 2016 | 2017 |
| No. of patients discharged on clozapine (%) | 225  (18.8%) | 218  (19.5%) | 227  (19.2%) | 176  (15.4%) | 205  (17.6%) | 211  (19.5%) |
| No. of patients discharged | 1194 | 1120 | 1185 | 1143 | 1165 | 1081 |

^1^KSPH = Kai-Syuan Psychiatric Hospital

Supplementary Table S6. Rate of augmentation with a second antipsychotic among patients discharged on clozapine, 2006-2017 (Only patients discharged from KSPH^1^ were included in the analysis)

| Year | 2006 | 2007 | 2008 | 2009 | 2010 | 2011 |
| --- | --- | --- | --- | --- | --- | --- |
| No. of patients discharged on clozapine + a second antipsychotic (%) | 30  (22.4%) | 51  (35.7%) | 62  (39.5%) | 72  (38.1%) | 78  (40.0%) | 73  (43.2%) |
| No. of patients discharged on clozapine | 134 | 143 | 157 | 189 | 195 | 169 |
| Year | 2012 | 2013 | 2014 | 2015 | 2016 | 2017 |
| No. of patients discharged on clozapine + a second antipsychotic (%) | 75  (33.3%) | 80  (36.7%) | 100  (44.1%) | 88  (50.0%) | 100  (48.8%) | 92  (43.6%) |
| No. of patients discharged on clozapine | 225 | 218 | 227 | 176 | 205 | 211 |

^1^KSPH = Kai-Syuan Psychiatric Hospital

Supplementary Table S7. Percentage of patients discharged on clozapine, 2006-2017 (Only patients discharged from TYPC^1^ were included in the analysis)

| Year | 2006 | 2007 | 2008 | 2009 | 2010 | 2011 |
| --- | --- | --- | --- | --- | --- | --- |
| No. of patients discharged on clozapine (%) | 107  (14.6%) | 144  (17.2%) | 164  (16.5%) | 153  (17.3%) | 168  (17.4%) | 176  (19.4%) |
| No. of patients discharged | 732 | 836 | 996 | 884 | 963 | 908 |
| Year | 2012 | 2013 | 2014 | 2015 | 2016 | 2017 |
| No. of patients discharged on clozapine (%) | 185  (20.0%) | 177  (19.8%) | 193  (21.6%) | 170  (19.7%) | 181  (21.1%) | 181  (20.7%) |
| No. of patients discharged | 923 | 895 | 892 | 861 | 859 | 876 |

^1^TYPC = Taoyuan Psychiatric Center

Supplementary Table S8. Rate of augmentation with a second antipsychotic among patients discharged on clozapine, 2006-2017 (Only patients discharged from TYPC^1^ were included in the analysis)

| Year | 2006 | 2007 | 2008 | 2009 | 2010 | 2011 |
| --- | --- | --- | --- | --- | --- | --- |
| No. of patients discharged on clozapine + a second antipsychotic (%) | 16  (15.0%) | 19  (13.2%) | 28  (17.1%) | 32  (20.9%) | 45  (26.8%) | 48  (27.3%) |
| No. of patients discharged on clozapine | 107 | 144 | 164 | 153 | 168 | 176 |
| Year | 2012 | 2013 | 2014 | 2015 | 2016 | 2017 |
| No. of patients discharged on clozapine + a second antipsychotic (%) | 32  (17.3%) | 46  (26.0%) | 46  (23.8%) | 60  (35.3%) | 51  (28.2%) | 50  (27.6%) |
| No. of patients discharged on clozapine | 185 | 177 | 193 | 170 | 181 | 181 |

^1^TYPC = Taoyuan Psychiatric Center
